# Metal Ions in Biological Systems, Volume 35: Iron Transport and Storage Microorganisms, Plants, and Animals

**DOI:** 10.1155/MBD.1998.262a

**Published:** 1998

**Authors:** Astrid Sigel, Helmut Sigel


					IN
METAL IONS IN BIOLOGICAL SYSTEMS
VOLUME 35
IRON TRANSPORT AND STORAGE
MICROORGANISMS, PLANTS, AND ANIMALS
Edited by Astrid Sigel and Helmut Sigel
Marcel Dekker, New York, 1988
775 pages, ISBN: 0 8247 9984 4
Volume 35 of Metal Ions in Biological Systems, entitled Iron Transport and Storage in
Microorganisms, Plants, and Animals, offers a comprehensive and timely account of this
fascinating topic by 35 distinguished international authorities.
In 18 stimulating chapters, this volume highlights first the biological cycling of iron in
oceans, followed by accounts of the transport of iron in microorganisms, fungi, and plants,
the roles and properties of siderophores, and the regulation of iron transport and uptake in
animals, plants, and microorganisms; it evaluates the structure and structure-function
relationships of ferritins and considers their uptake, storage (mineralization), and release of
iron; it examines transferrin and its receptor, the homeostasis of iron, especially in humans,
and the effect of other metals on iron transport, and it terminates with a chapter about the
challenge to create iron chelators for clinical use.
With more than 2200 references to assist further research, Iron Transport and Storage in
Microorganisms, Plants, and Animals is an essential resource for scientists and students in
many disciplines, including bioinorganic, inorganic, and coordination chemistry;
biochemistry; biophysics; molecular biology; enzymology; pharmacology; physiology;
clinical chemistry; nutrition; toxicology; and environmental sciences.
262

				

